# The impact of cancer expectations on psychological responses following a cancer signal detected result in asymptomatic multi-cancer detection (MCED) testing

**DOI:** 10.1038/s41416-025-03180-5

**Published:** 2025-09-09

**Authors:** Laura A. V. Marlow, Ninian Schmeising-Barnes, Jo Waller

**Affiliations:** Centre for Cancer Screening, Prevention and Early Diagnosis, Wolfson Institute of Population Health, Queen Mary University of London, London, E1 1HH UK

**Keywords:** Human behaviour, Risk factors

## Abstract

**Background:**

Multi-cancer detection (MCED) blood tests have the potential to screen for early-stage cancers. Understanding how people experience an MCED cancer signal result is vital prior to any future implementation. We explored experiences in a trial context.

**Methods:**

A subset of 41 participants in the NHS-Galleri trial (NCT05611632), with a cancer signal detected result, were interviewed. We selected: 20 participants with cancer found (self-reported) and 21 without cancer (following tests). Transcripts were analysed thematically.

**Results:**

Expectations of cancer played a pivotal role in emotional, cognitive and social responses, and were influenced by participants’ experiences of health and symptoms. While the cancer signal was often unexpected, the predicted cancer signal origin made sense when consistent with family history or health issues. During the period of diagnostic uncertainty, views of healthiness or lack of family history were sometimes used to self-reassure. For some, a cancer diagnosis was unexpected; for others, expectations of cancer had gradually increased. For those without cancer, believing that it could be present affected their sense of reassurance.

**Discussion:**

Information about the meaning of a cancer signal will be needed if MCED screening is implemented. Patient support could be designed around their expectations of cancer at each stage.

## Background

Multi-cancer detection (MCED) tests are a new potential approach to cancer screening. These tests look for a ‘signal’ associated with cancer using samples such as blood or saliva [1, 2]. Unlike existing single cancer screening (e.g. for breast, cervical or bowel cancer), an MCED test would indicate cancer in one of many possible sites. The novelty of this approach has implications for how people would make sense of their screening result and how they interpret diagnostic resolution [3].

Research exploring the psychological impact of cancer screening suggests that those who have ‘abnormal’ results often experience higher levels of anxiety, worry and concern about cancer, particularly in the short term [4–8]. Many people who take part in screening attend because they feel it is a good way to look after their health generally, rather than in pursuit of a diagnosis [9]. This accurately represents the purpose of screening which, in the vast majority of cases, provides the person with a ‘normal’ result (i.e. no indication of cancer or increased cancer risk). However, since people attending screening do not anticipate abnormal results, receiving these are unexpected. Studies suggest that a general feeling of being ‘healthy’ means people do not consider themselves to be candidates for cancer and test results that suggest the possibility of cancer are therefore unexpected [10, 11]. These feelings can extend beyond the initial result communications, with evidence that a screen-detected cancer diagnosis is perceived differently to one following symptom investigation. Unlike a diagnosis following symptoms, which is accompanied by validation and relief, a diagnosis following an abnormal screening result comes with shock and disbelief [12, 13].

This paper describes our interpretation of the role that cancer expectations can play in how people make sense of a cancer signal (positive) result following screening with an MCED test and how this impacts their narratives regarding cognitive and emotional responses, and social interactions throughout diagnostic investigation.

## Methods

### Design and methodological approach

This research explored the experience of having a cancer signal detected test result within the NHS-Galleri trial (NCT05611632) [14], as part of the Psychological Impact of the Galleri Test (sIG(n)al) project [15]. As researchers with backgrounds in psychology, we consider ourselves to be critical realists in our approach to understanding attitudes, beliefs and experience of cancer screening and cancer diagnoses. We consider these concepts to be complex and influenced by social and political contexts. We therefore believe that people will perceive and experience having a cancer signal detected in different ways. With this in mind, we believe that people make meaning of their reality and that we can capture, interpret and explain multiple participant realities using a non-positivist (or ‘Big Q’) qualitative approach [16]. We have reported the methods and results of this study following the consolidated criteria for reporting qualitative research (COREQ) checklist [17].

### Trial context

The NHS-Galleri trial is a randomised controlled trial of asymptomatic adults aged 50–77 years, with no history of invasive cancer in the 3 years prior to enrolment [14]. Trial enrolment ran August 2021-July 2022. All participants provided a blood sample. Participants in the intervention arm had their blood samples tested with the Galleri® test (GRAIL, Inc., Menlo Park, CA, USA), an MCED test which looks at methylation patterns on cell-free DNA, detecting a cancer signal and predicting the tissue type or organ associated with the signal (cancer signal origin; CSO). In some cases two CSOs were predicted. Participants who had a cancer signal detected were informed by phone by a research nurse. They were told the predicted CSO(s) and were referred into an appropriate NHS cancer diagnostic pathway (depending on CSO). All trial participants were invited to return for two repeat screening rounds at yearly intervals, unless cancer was diagnosed.

### Participants

Participants who had a cancer signal detected indicated their interest in being interviewed within a questionnaire sent 6 months after receiving their result (return rate = 72%). Of those who returned the 6-month survey, 68% indicated interest in being interviewed. By this point, participants were expected to have completed follow-up diagnostic tests and know whether cancer was found. We planned to interview 20 participants who self-reported a cancer diagnosis after further tests and 20 who self-reported that no cancer was found (at the time of the questionnaire). The planned sample size was pragmatic, taking the expected information power of the sample into account [18]. Within each of these groups, roughly equal numbers of participants were selected to be male/female; 50–65/66–77 years old; and high/low deprivation. Deprivation was assessed using the Index of Multiple Deprivation (IMD), with an IMD deciles 1–5 considered low deprivation and deciles 6–10 high deprivation (see [19]). Those selected for interview were sent an information sheet and were contacted 1 week later to confirm interest and arrange an interview. We made a maximum of three attempts to contact selected participants by telephone, with a voice message left on the final attempt. Informed consent was obtained before the interview. Ethical approval was granted by the Wales Research Ethics Committee as part of the NHS-Galleri trial (Ref 21/WA/0141).

### Procedure

Interviews were carried out by telephone, face-to-face or on Microsoft Teams (depending on participant preference) and were audio-recorded. Most were carried out by LM, with a few by NSB. Both researchers had training and experience in qualitative methods. The researchers were not known to the participants before the study took place. No-one else was present for the interviews. Following the interview, participants were given a £40 voucher.

Interviews were semi-structured and followed a topic guide developed by the research team with support from a Patient and Public Involvement (PPI) panel (available on OSF: https://osf.io/cau6p). The topic guide included reasons for interest in taking part in the NHS-Galleri trial (not the focus of this paper), experience of receiving results, secondary care work-up, information needs and reflections on the overall experience. The order of the discussion was driven by the participant. After the first three interviews, the topic guide was revised so that participants were invited to explain their experience in their own words at the beginning of the interview. This helped to encourage open discussion and highlighted the aspects that were most prominent to each participant.

### Analysis

Recordings were professionally transcribed verbatim and identifying details were removed. NVivo 1.7 was used for data management. We used Reflexive Thematic Analysis (TA) [20]. This method focuses on coding and developing patterns (themes) within the data. Coding is organic and flexible, and theme development moves beyond the superficial with in-depth engagement from the researchers. There are six, often overlapping, phases of Reflexive TA, with movement backwards and forwards between phases. In brief, LM familiarised herself with the transcripts by reading and re-reading each one, coded the data by labelling sections of the transcripts and then used mind-mapping to organise these codes into initial themes. Themes were reviewed/developed, discussed in detail with co-authors and refined during report writing. This paper describes themes that related to making sense of the experience of receiving a cancer signal test result. We will report service-related themes that represent experiences in the diagnostic period in a separate paper.

### Reflexivity

LM had previous experience of facilitating interviews in cancer screening but not with people who had experienced abnormal results or a cancer diagnosis. At the time of data collection LM, NSB and JW were a senior researcher (PhD), research assistant (MSc) and reader in cancer behavioural science (PhD) respectively. All are female and have training in health psychology. LM wrote a reflexive statement before data collection reflecting on her pre-existing conceptions and kept reflexive notes during data collection and analysis.

## Results

We approached 53 of those who were interested and 41 were interviewed (12 had changed their mind or could not be contacted). Participant characteristics are reported in Table 1. Interviews lasted 22–80 min (mean = 43 min). Median time from receiving result to participating in an interview was 7.1 months. CSOs were wide ranging and of the participants who were diagnosed with cancer a broad range of cancer types were self-reported; 12 different cancer types across the 21 participants who self-reported a cancer diagnosis.

**Table 1:** Characteristics of interview participants (*n* = 41).

	Overall (n)
**Gender**	
Male	20
Female	21
**Age**	
≤64	14
65-74	22
≥75	5
**IMD Quintile**	
1 (most deprived)	8
2	8
3	6
4	11
5 (least deprived)	8
**Ethnicity**	
White British	37
Mixed ethnic background	1
Any Asian background	2
Any Black background	1
**Marital Status**	
Single, never married	3
Married, in a civil partnership or living together	29
Divorced or separated	6
Widowed, not remarried	3
**Highest level of education**	
Finished school at or before the age of fifteen	4
CSEs, O-levels or equivalent	9
A-levels or equivalent	2
Further education but not a degree	12
Bachelor’s degree or equivalent	10
Further degree e.g. masters or PhD	4
**Self-reported cancer diagnosis**	
Yes	21
No	20
**Interview mode**	
Telephone	36
Face-to-face/Teams	5

Interviews reflected participants’ journeys from (i) receiving a cancer signal detected result, through (ii) the diagnostic testing period, to (iii) the diagnostic outcome (cancer found or not found). The results section has been structured according to these three stages. Within each stage we have presented themes that summarise participant reflections regarding cognitions, emotions and social interactions. Supporting quotes are reported in the text and in Table 2. Expectation of cancer as a possibility was a higher-level (latent) theme that appeared to cut across different aspects of participants’ narratives.

**Table 2:** Supporting quotes for each theme.

Theme/Subthemes	Quotes
***Receiving a cancer signal result***	
Shock and surprise	*“I mean, it was kind of a heart sink moment, but at the time, I don’t remember feeling startled. I just remember that I think the world’s just dropped out at the bottom”* (P1, cancer found) *“I must admit, I was sort of, thrown back… the results obviously, when you get them in the first instance, it’s a shock. Your head just goes scrambled, you can’t even think straight. You don’t know how to put one foot in front of the other”* (P16, cancer not found) *“Yeah, I suppose naturally you feel quite shocked. And it’s quite a numbing feeling really. Because it’s something you never want to hear… it was just a lot to take in really. It didn’t feel real, because it’s just something you just didn’t think you would hear really”* (P22, cancer found) *“I did get a little bit upset when I was called back to say there was two markings or signals whatever you call them for cancer. Obviously it was just a bit of a surprise, really. But I understand you have to do it, and that’s what it’s all about”* (42, cancer not found) *“So you’re shocked, you’re thinking, I could do without this. But, just took it in my stride and got on with it”* (P5, cancer found) *“No, no I thought big, strong [NAME], he’ll be alright. And then when the phone call comes, I’ve been hit with a sledgehammer, to be honest”* (P14, cancer found)
Making sense of the result	*“I didn’t have any symptoms. There was nothing to indicate there was anything there. And that’s what was the shock was. The fact that I thought I was perfectly healthy. And then I got a phone call saying that there was a potential, that was a shock”* (P27, cancer found) *“So no, I didn’t expect to hear anything because, you know, I’ve had no symptoms you know. So, yes, it was a bit of shock. I’ll be honest”* (P33, cancer not found) *“I was quite surprised really at how shocked I was when the results came back and it showed that I had cancer signals in two areas. And to be honest, it really did take the wind out of my sails, if you will… I think when it actually, when you get that letter that actually says signals have been detected in two areas, it is quite a shock when you don’t feel unwell in any way and you have no other outward signs that something could be wrong”* (P36, cancer not found) *“I must admit it was a little bit of a shock. Because, I mean, I don’t know what to expect or what not to expect. I mean, I felt healthy enough, you know, in, how I feel, how I work and all that, You know, I wasn’t lethargic or anything, you know, I didn’t think anything was actually wrong, because I’m pretty fit and healthy really for an old bloke. So it, like I say, it was a little bit of a shock but then you just gotta get on with it and just hope for the best”* (P38, cancer not found) *“So I I thought, Well I don’t look sick. I don’t look, you know, I don’t feel sick. Where is this coming from? You know what I mean? And I thought it can’t be right, you know”* (P20, cancer found) *“And I was just thinking, he’s got that wrong because I’m quite healthy you know, as you do [laughing]. So it was a bit unbelievable to be quite honest because I didn’t expect it and when he did tell me that they’d found something, I didn’t believe it anyway. But as it happened, he was right”* (P3, cancer found) *“I mean, I was in a sort of, you know, I couldn’t quite believe it at first, you know, …I think I was sort of in denial thinking, you know, not me, you know, I’m healthy etcetera, and I don’t have any symptoms at all. I, you know, I just couldn’t get my head around it, really”* (P30, cancer found) *“I’d had bowel cancer [screening], I’d done that test as well. And my expectations were that it would all be negative. I never thought in a million years, I think, that it would be such a positive result… I dismissed it from my mind, actually. I hadn’t -- I didn’t worry about the results. It hadn’t entered my head that anything would be positive because any tests I’d ever had in the past had always been negative. And I’m somebody that’s never been ill. I’m not -- all my life, I’ve enjoyed good health. So I didn’t think about it at all as being something to worry about.”* (P6, cancer found) *“At 76 years of age, expectations in life has taken on a new meaning, really”* (P31, cancer not found) *“Well, if it is a 50/50 chance, when you get to 78 you can expect all kinds of signals”* (P4, cancer found)
Coherence of the predicted Cancer Signal Origin	*“I was surprised. I was very surprised. I was surprised that there was a positive result for cancer and even more surprised at where it was. Because I hadn’t, you know, I hadn’t had any bleeding or anything.”* (P10, cancer found) *“No, because I was having no side effects, I was having no symptoms. And if I was going to get anything else, I would have thought it would have been in a similar sort of place to where I got it before, you know, like, skin, rather than internally. I was just, I was prepared for externally. But not for internally, does that make sense?”* (P20, cancer found) *“I have, you know, I’ve done these home bowel tests twice in the past, which I’ve been quite happy to participate in those, and send them off, and they’ve always come back negative, so. And I’ve had no symptoms which would suggest any problems”* (P25, cancer found) *“And I’d had a PSA test on the prostate a couple of months earlier and the PSA reading was right down, like three, or something like that. But it is slightly swell, I forget what they call it, you know, it’s a benign hyboplastic, or something, I don’t know, I can’t remember the proper wording. But so, you know, when they said me prostate, I thought, Oh perhaps something’s happened with that”* (P13, cancer not found) *“And by coincidence, I had been to the GP shortly before that, with a slight pain in my stomach.… this was before I got the positive result. So the two sort of came together, really”* (P22, cancer found) [describes experience of suffering with stomach problems for years as a result of a bacterial infection and recent conversation with GP about symptoms and possible referral to gastroscopy] *“… the markers were suggesting the stomach, when she mentioned the stomach, there was sort of a light bulb moment, and I thought, yeah, if there’s anything going to be happening, it’s going to be the stomach. So, I wasn’t entirely thrown sideways when she mentioned that”* (P25, cancer found) *“I misunderstood it. Or well, the way I understood initially was that I could have cancer in both my kidney and my pancreas gall bladder. That’s the way I understood it, first of all… And then it was explained to me, the test is looking for primary cancer, and the chances of having two primary cancers at the same time was very, very unlikely.”* (P22, cancer found)
Implications of the result for cancer risk	Meaning of the result: *[Interviewer: And what did you think the markers meant?] “That there was some sort of, either, some sort of cancer in my system somewhere. Or the early signs of it”* (P19, cancer not found) *“And I thought, well, there’s nothing, there’s obviously nothing wrong with me. But obviously there may be something there that’s been picked up, but it might be erroneous. The signal might be erroneous”* (P27, cancer found) *[Interviewer: Okay, and how did you feel while you were waiting for the PET scan?] “Again, I still wasn’t worried because I’m thinking, it might still come back with nothing to worry about. No, I didn’t worry*” (P18, cancer found) *“It could be a possibility that I could develop cancer”* (P18, cancer found) *“I found that a bit confusing… how could you have a positive signal but haven’t got cancer”* (P22, cancer found) *“as soon as I got the, as soon as I got the letter and the telephone call from the lady in London saying, We think, well, we’ve picked up a cancer track, I was laughing me head off. Okay, fair enough be, you’re dead, you’re dead, you know that’s the first thing that hits anybody I think”* (P34, cancer not found) *“Well immediately I thought, I’ve got cancer … and then I kind of calmed down a bit after that and I thought, you know, it could easily be a false positive”* (P10, cancer found) *“I went from, This is all right, to, Oh the worst is about to happen”* (P28, cancer not found) *“I just thought, Oh, gosh, you know, this has to be investigated, and it may show something, and it may not show something, but you tend to focus on the negative really I think rather than -- and, you know, you do tell yourself, Look, it could be okay, try not to worry about it too much until you’re actually told that there is something definite that you need to take action on. And that’s easy to say, but it’s not quite so easy to do in reality.”* (P36, cancer not found*)* Self-reassurance: *“I thought, right, okay, I’m not feeling ill. So therefore, hopefully, it’s treatable, if I’ve got it.”* (P28, no cancer found) *“I didn’t feel any pain, I wasn’t losing weight or anything like that, so nothing suggested to me that if there was a cancer there, from what I understand about some cancers, it didn’t suggest to me it would be advanced anyway”* (P25, cancer found). *“And then when they said they’d found something, it was a bit disconcerting, but I wasn’t awfully worried because I really didn’t have a lot of, apart from the bowel problems I’d had, I really didn’t have a lot of symptoms of anything”* (P19, cancer not found)
Social implications	“*I think my wife sort of felt, or got hit about it, or was took it more, worse than I did, I think, because it was going to be sort of this possible sort of sudden or complete change in your lifestyle*” (P25, cancer found) “*I decided, really, that I didn’t want to tell anybody about it but all the time it was going through my mind. How do I actually start this conversation if, you know, if I need to*” (P33, cancer not found) *“It came at, rather at that time, because my son in law had been diagnosed with cancer…. So, obviously it struck home a little bit more and then when I got the phone call, I was in the kitchen like I am now, speaking, and my wife were in the lounge. So then after when I tell her that I’ve been told I got cancer. Now whether it were a little bit of the shock of how it was explained to me, I don’t know, but I just took it that I got cancer.”* (P9, cancer not found) *“it was only sort of five months since I’ve lost my partner in very horrendous circumstances… So I was very vulnerable as it were and emotional, so I think that obviously didn’t help”* (P42, cancer not found) *“I regarded as highly inconvenient because we’ve got nine grandchildren… and we were putting in a lot of support. I thought I really haven’t got time to die”* (P28, cancer not found)
***Having diagnostic tests***	
Concern about cancer	*“I’m not a negative type, so I was quite positive about it, you know, so, you know, at the end of the day obviously it’s a worrying time for us both, so we -- you know, it’s we have to investigate it and find out what was going on. And that’s what we did.”* (P21, cancer found) *[Interviewer: Were you worried while you were waiting for that appointment?] “No, not at all. Because again, I thought it was just a false signal. I just never even considered it.”* (P27, cancer found) *“But I found it didn’t really sink in, and it was a complete mixture of emotions, really. It was obviously a bit of worry. You feel anxious and you want more and more information. But yeah, I didn’t feel, yeah I was worried and anxious, but I wasn’t in a complete, terrible state, you know, I was still all together”* (P22, cancer found) *“Yeah, I wasn’t jumping with glee certainly, but sort of I went into a bit of a reset, I guess, I thought, you know, Right, okay, this is a potential real problem there, which has been identified. And so I was looking forward, hesitantly I suppose, for the initial tests to find out what it was and what the long term implications of it was going to be. So I was, yeah, I was a little bit nervous in that initial period.”* (P25, cancer found) *“The first thing I did was I bought myself a new watch. New wristwatch, luxurious new wristwatch, this will make you laugh. So I could watch my life ticking away”* (P34, cancer not found) *“Until someone turns round and actually says you’ve got it, there’s no point worrying, is there… so I just went on as normal”* (P15, cancer not found) *“I think I shocked her actually, it didn’t bother me in the slightest. It’s, something’s got to get you, and there’s plenty of, a bunch of stuff out there that is far worse. Yeah, so that was it really”* (P34, cancer not found) *“I’m quite a happy-go-lucky, sort of person. Just carry on and just deal with what you’ve got and you can’t change things about what you’ve got. So I don’t worry about what I’ve got, what I haven’t got, what could be, what couldn’t be, I was going, Well, that’s it, end of story. Do you know what I mean?”* (P38, cancer not found)
A pressure to discuss with others	*“My family, I didn’t really tell because, obviously being told -- as in the biopsy in December, and over Christmas time, I didn’t mention it to the children at all because I don’t want to ruin everyone’s Christmas”* (P18, cancer found) *“I wasn’t going to tell my family until I kind of had got an appointment, and had the investigations, and they knew what was what. But the problem was, I just thought it’s too complicated, because I’d have to take time off work. I’ve got to get up to [NAME OF HOSPITAL]. I’d have to have scans… and I just thought, to have to lie to my family about I don’t know how many appointments would just be ridiculous”* (P1, cancer found) *“I don’t like being asked about it, because then it’s on your mind all the time and I’d rather it not be in the forefront of my mind, I’d rather be at the back of my mind. Just wait and see and you know, rather than that be, have you got your results back. No No No. Then you start worrying and all that lot, and I just don’t want to worry”* (P38, cancer not found) *“So then we managed to obviously tell the children what had been going on, and obviously got a severe telling off. Because, you should have told us, you should have done this. We said, Well, we didn’t want to upset you lot, or the grandchildren, or anyone else, if there was really nothing to worry about and destroy your Christmas”* (P16, cancer not found)
***Following the diagnostic outcome: Cancer found***	
Feeling prepared for a diagnosis	*“it was obvious that there was something not right. I can see it on the screen. I said to the specialist who was doing the test I said, That doesn’t look good? And he said, No, it’s not good”* (P27, cancer found) *“But he showed me the stomach area, and as soon as he showed me the photograph, you could see that whatever was there, it shouldn’t have been there”* (P25, cancer found) *“And I must say, at that point, their attitude had changed completely to me. And I thought, shit, they’ve seen something”* (P1, cancer found) *“Dr [NAME] was speaking to the nurse and said, if it’s such and such it’ll be tested here, but if it’s Non-Hodgkin’s Lymphoma or if we think it is, it will go to [CITY]. So I knew there was something wrong when I kept ringing up and they said, It’s going to [CITY] for further tests. So I knew then that it was worse than what was first thought”* (P5, cancer found) *“[After the biopsy] I went and stayed at my sister-in-law’s and that was when I really thought -- began to think I had cancer. Because we looked at the notes that they send you home with, with what they’ve actually done, and it said endometrial suspicious. And so that was the point I thought this really could be cancer”* (P10, cancer found) *“So it gives you time to get your head around it, yeah, so I think mentally the diagnosis of cancer was a drip-drip. So It wasn’t a horrendous shock to me because I’d sort of been given a 50/50 chance right from the start of it being cancer.”* (P26, cancer found) *“Because the test was so specific and targeted, um, when they did tell me the results I think I was more prepared for it than I would have been if they just said there’s a potential for you to have cancer.”* (P3, cancer found)
Still not expecting cancer	*“And then they told me there that I had cancer. And honestly, I couldn’t believe it. I suppose I was just in denial about it but I couldn’t believe it when she said, Yeah, it’s cancer and you’re going to have chemotherapy and surgery and everything.”* (P11, cancer found) *“They basically sat us in the room said, there is a tumour and it’s likely to be cancerous, so you will need an operation. So that was the, sort of the worst, one of the worst parts of it because obviously you don’t expect, I didn’t expect anything in terms of that to be the result of the trial. So it was a bit hard again. It was a bit of a shock when I saw the tumour on the screen”* (P27, cancer found). *“I mean, even though you’re kind of expecting it still comes as a bit of a shock, and I remember leaving the hospital, I did shed a few tears. Shed a few tears as I was walking out of the hospital”* (P22, cancer found) *“I’m not a sicky person. And my default attitude is always positive and healthy, so yeah, this was just, I’ll do that. But I never, never expected there to be anything. And I confess, right up until I saw the photos from the colonoscopy, I was in denial.”* (P35, cancer found) *“Well I went along for the test and I seriously didn’t think I had cancer or there was -- I had no symptoms whatsoever. So it came as a surprise when the -- I got the phone call to say there were markers for cancer. And even then I thought it could be a false positive. And so it wasn’t really until after the biopsy that I seriously thought I could have cancer.”* (P10, cancer found) *“I was getting very, very tired all the time. We think possibly I did have a symptom I didn’t know about… my work load had got busier and I think I put it down to that., but thinking about it now, chances are, I possibly did have the symptoms but I’d, me being me, got through it.”* (P32, cancer found)
***Following the diagnostic outcome: Cancer not found***	
Feeling reassured	*“… it gave me the result, you know, that I’m 99 per cent sure would come back anyway, that there was nothing wrong and that.”* (P40, cancer not found) *“In my heart, I knew anyway. I didn’t think I had anything because I had no signs of anything beforehand. But it was a relief because you never know, you never know.”* (P40, cancer not found) *“And it’s reassuring. I’m very reassured that I’ve had my full body scan and nobody has come up and said, Oh, you’ve got something here. That is untoward.”* (P31, cancer not found) *“Because a lot of people have had results and they’ve come back that they’ve actually got cancer. But I’ve got these results and nothing is showing. So I’m sort of in between the two really.”* (P29, cancer not found) *“I didn’t leave feeling okay, that’s fine. I have nothing to worry about. I was left feeling a bit – it just felt a bit inconclusive”* (P41, cancer not found). *“I was reassured enough. Yes, but I don’t think in this day and age, and when you get to a certain age, I’m 70, that, you can never be absolutely sure of anything. So it was in no way negative. I wasn’t thinking negatively about it. I was just just relieved and I suppose I’m quite, a practical person. So you can never be sure of things in life put it like that.”* (P42, cancer not found) *“Well, you know, I am over sixty, I’m mid sixties now. So obviously, at some point you are going to face ill health and you know, to look to things in the future. So maybe I’ll deal better with things in the future, having been through this process, maybe it will help me to deal with things better as they arise.”* (P36, cancer not found)
Meaning of the result	*“Well, if you haven’t found anything, why were the flags there? You know, what were they saying? Is this something for the future or -- I mean, obviously, I don’t understand the blood test in that much depth, so I don’t know what they’re saying, why they’re saying what they’re saying”* (P19, cancer not found) *“everything’s fine now. Is there a higher risk? In future is an indicator that you know, am I my more likely to get cancer than someone else.”* (P33, cancer not found) *“I think just maybe a clearer explanation as to what it is in the blood that you’re looking for or had found, and what that can mean. Or if it does mean nothing, as in there is nothing found, why was the signal there in the first place? I think that’s more the thing. You know, could it mean potential for the future that something could be found? Or could it mean that something has been found in the past? You know, that is indicating some anomaly.”* (P41, cancer not found)

### Receiving a cancer signal result

#### Shock and surprise

Participants described feelings of shock and surprise when told that a cancer signal had been found. This was described as a *“heart-sink-moment”* and participants felt *“taken aback”*. Participants also described a feeling of not knowing what to think: *“your head just gets scrambled”, “you can’t even think straight”*. Shock was influenced by the lack of expectation that cancer was a possibility: *“never in my wildest, you know, expectations thought it was going to come back with a positive”* (P20, cancer found).

#### Making sense of the result

Perceptions of existing ‘good’ health and a lack of symptoms conflicted with a test result that raised the possibility of cancer: *“I wasn’t feeling anything, I didn’t feel ill, I wasn’t losing weight. And I thought, well, there’s nothing, there’s obviously nothing wrong with me”* (P27, cancer found). Broader perceptions of the self as a healthy person, who exercises or has been screened recently were also drawn upon: *“It hadn’t entered my head that anything would be positive because any tests I’d ever had in the past had always been negative. And I’m somebody that’s never been ill. I’m not -- all my life, I’ve enjoyed good health”* (P6, cancer found). For some, the finding was more easily incorporated into existing feelings of health and symptom experience: *“So before I got the phone call from the trial, I’d been to see a doctor who thought I had a gynae problem and the two things sort of came together”* (P11, cancer found). Some participants reported a previous cancer (more than 3 years before) and the result was interpreted in light of this.

A broader view of sense-making was evident in surprise about the result. For example, several participants in their 70 s acknowledged that being older came with the potential for disease: *“I was mildly surprised, but not very, very surprising… because of my age, I wasn’t surprised something might have [been] found” (P21, cancer found)*. Family history of cancer also made it easier to make sense of a cancer signal result: *“I suppose I were a little bit biased because as I’ve said, both my parents have died of cancer and it has been said in the past that it could be hereditary. So I suppose I were half expecting to be given a positive result” (P9, no cancer found)*.

#### Coherence of the predicted cancer signal origin (CSO)

While the cancer signal result was unexpected, the CSO made sense if it fit with the participant’s family history, any recent unexplained symptoms they had been having or another health issue related to that organ/tissue. Previously clear screening tests for the organ/tissue sometimes led to confusion: *“And, the one for the anus came back as a bit of a surprise because I had maybe two or three weeks before, done a poo sample, you know, to be tested for cancer and that had come back clear. So I was quite shocked about that, and it took me a little while to sort of think, you know, that can’t be right.”* (P20*, no cancer found*). This in fact indicates a misunderstanding of the purpose of bowel screening but highlights how understanding of existing screening results can influence interpretation of a predicted CSO within MCED screening. For some, the CSO was biologically unexpected, because the organ had previously been surgically removed (e.g. cervix or gallbladder); *“how can I have uterine cancer if I don’t have a uterus? So is that a mistake?”* (P1, cancer found). CSOs for less familiar tissues (e.g. ‘lymphoid lineage’) were more difficult to incorporate into existing understanding of a cancer, with terminology hard to interpret: *“I couldn’t quite remember the exact terminology, so I think I did a big bit of google. And I just thought a lot of just millions of things you could be looking at here and quite frankly, chances are you’re just scaring yourself and looking at the wrong stuff.”* (P33, no cancer found).

#### Implications of the result for cancer risk

Participants understood the meaning of the result in different ways. Some recalled thinking that the result was a cancer diagnosis: *“She said, you’ve been diagnosed with the early stages of cancer”* (P5, cancer found); others felt it was likely to be a ‘false positive’: *“50% of people who get this phone call don’t have cancer, so I just thought I’d be one of them”* (P11, cancer found)(Note: this is consistent with what participants were told by the nurse who was following a script that stated; ‘*About half of the people who have a possible cancer signal detected will be found to have cancer during the follow-up tests’*). There were also examples of people who felt their result might indicate cancer with varying degrees of certainty ranging from ‘a possibility’ to ‘likely’. Assessing what the result meant for them was a process and some described how their expectations and feelings associated with these varied over the coming days: *“So I was sort of quite calm to begin with then within a couple of days, I was totally convinced I was about to die”* (P28, cancer not found). Some participants who recalled being told that the test had reported two CSOs interpreted this as meaning that they could have cancer in both locations or that the cancer had metastasised.

For those not expecting cancer, views of healthiness, lack of family history or results from other recent screening tests were sometimes used to self-reassure that the test result did not indicate cancer or if it did, the cancer must be treatable: *“I wasn’t particularly worried about it because I don’t know whether it helps or not, but I did have a regular bowel test, and that has shown up as nothing”* (P31, no cancer found).

#### Social implications

Managing relationships and disclosure during this period was widely discussed. Most described telling their partners, recalling worry and upset in these conversations: *“I think my wife were like me, a little bit numb by it. Probably a little bit more upset than I were.”* (P9*, no cancer found*). Some wanted to avoid telling others or not knowing how to. This was largely to avoid causing worry but also because they did not want to be asked about the outcome or because they did not think it would be cancer: “*I didn’t tell my husband or any of my family because I just thought, well when I go there it will be a mistake. So there’s no point getting everybody stressed for nothing. So I didn’t actually tell anybody at all that I’d had the conversation with him or with the nurse”* (P3*, cancer found*). While considering the possibility of cancer in the days following the result, several participants reflected on how a diagnosis would fit within their current circumstances: *“two days prior to this, we just learned that we were going to be grandparents again. And I think that made it worse really. Because, you know, I sort of have myself thinking, What if I’m not here to see this baby born?“* (P36*, no cancer found*).

### Through the diagnostic testing period

#### Concern about cancer

Some participants described worry during the diagnostic period, and some described more intense concern. In particular, those who imagined their potential future as including a cancer diagnosis felt they needed to prepare for this. For one participant these thoughts were intrusive and impacted sleep and life: *“And that sort of keeps playing on your mind all the time, so I think I must have lost quite a bit of sleep over that, because your brain starts going round saying, You’ve got cancer. And then I’m looking, I’m 67 years old, got grandchildren, etcetera, and I’m looking at, I’ve gotta put my house in order. I’ve got to check my insurance policies. I’ve got to check this, I’ve gotta make sure the bank accounts are done and make sure everything…So over night time you’re trying to go to sleep thinking that, you possibly could have cancer, and you need to get everything sorted out. So I was waking up at three o’clock in the morning, and trying to get everything done.”* (P16*, no cancer found*).

Others described being matter of fact about the process, not worrying about cancer until it was diagnosed or expecting it would not be found; *“I try not to think of things, to not to get too bound up with the thoughts of it rather than wait and deal with what is, rather than what I think it might be, you know?” (P26, cancer found).*

#### A pressure to discuss with others

In some cases, the need for multiple visits and prolonged diagnostic periods raised difficult decisions about disclosing the potential for cancer to others. Participants described a conflict between not wanting to worry family members and not wanting to lie to them or needing to step back from commitments to attend tests (e.g. caring roles). This was particularly hard over events such as Christmas; *“We got to the point of my husband saying we’re going to have to say something because, you know, [daughter’s name] wanting to know if we could go this weekend, or that weekend… so in the end, we did tell them before the final letter comes through.”* (P28*, no cancer found*).

This was also the case for some in relation to work, where they needed time off. Some who told others and went on not to have cancer diagnosed regretted sharing their story: *“I went and told a few of my friends, and I think they were more upset than I was. And then six months later, I’m going to go back and tell them, Look, they can’t find anything. I’ve got to break the news to them, that quite probably upset them for nothing. That was the thing that really got me … if it comes back positive this year, I’ll keep me mouth shut. I’ll keep it to myself. I’m not putting other people through that again” (P34, no cancer found).*

There was also an example of someone being forced into disclosing when contact from the hospital was picked up by a family member: *“I kept it away from her until the point where a phone call came to the house … My daughter knowing absolutely nothing about it and her questioning, Why was I getting these phone calls? So we had to sit down with her and go through it all”* (P33*, no cancer found*).

### Following the diagnostic outcome

#### Cancer found

##### Feeling prepared for a diagnosis

For some, the experience of going through diagnostic tests, accumulating results and reading the context around them pointed towards cancer before a diagnosis was given. Participants looked for clues in their journey, describing how they knew it was cancer before officially being told. This included looking at notes, seeing scans that looked strange, being offered Macmillan nurse support, sensing a change in health-care staff attitudes or having tests sent away to other hospitals: *“…instead of the doctor coming out to greet me, there was Macmillan nurses and straight away I thought they’ve got the diagnosis, and it looks like it’s positive that I had cancer”* (P17, cancer found). Some participants described how having a cancer signal found and undergoing diagnostic tests meant they had gradually come to expect a diagnosis. This meant the cancer diagnosis itself was not such a shock. The prediction of a cancer site, followed by a consistent diagnosis was also described as helping to prepare for the diagnosis: “I*t was sort of in stages that I found it out. And so it was quite I think it was quite good how it was done, that it wasn’t, it wasn’t like a bomb dropping on me. It was just a little, a little bit at a time”* (P11, cancer found).

##### Still not expecting cancer

For most participants who had cancer found there was still a sense of shock. These participants had gone through the diagnostic process convinced that cancer was not there: *“I really, really didn’t think there was anything wrong with me…I kept sort of thinking about it and thinking it can’t possibly be right. And then I thought, Well it must be right because all these people can’t be wrong. It was a bit of a shock to the system to be quite honest”* (P3, cancer found).

Even after receiving a cancer diagnosis some reflected on the lack of symptoms or their perception of being someone who is healthy: *“I expected I would be showing some form of symptom or something like this, ‘cause they’re always saying, Look for the symptoms. But there weren’t any symptoms.”* (P4, cancer found). Other participants who had cancer diagnosed described symptoms they had experienced prior to the cancer signal result but had not recognised or attributed as being meaningful: *“I had absolutely no symptoms other than tiredness, which I would never have followed up on because I put it down to waking up every night, every hour, you know, so, yeah, I think I’d probably run out of steam”* (P35, cancer found).

#### Cancer not found

##### Feeling reassured

There was an overwhelming sense of relief that cancer was not found; *“once you get the okay bit, it’s like somebody lifts a big 20 tonne weight off your shoulders”* (P16, no cancer found). Participants described themselves as ‘lucky’ but some added things like ‘at this time’ suggesting some residual concern: *“Relieved and hope that the result was correct”* (P42, no cancer found).

One participant described how they had interpreted a recent illness in light of their experience: *“I have come down recently with quite a bad cold, I had COVID, all the rest of it, which is unknown for me to have anything like that. You know, I can’t remember the last time that I was -- really came down with anything. So when that happens, or when it’s happened, there’s still a little niggle … even though I know I haven’t, there’s still just occasionally this little, look is it like, is it going to be something in the future?”* (P33, no cancer found).

Some participants were not reassured by the news that no cancer had been found: *“It was quite unsettling afterwards, thinking well everyone said everything’s okay, but how do I know that? And how do they know that?”* (P41, no cancer found). Ongoing concern ranged from “a niggle” to outright frustration: *“the thing that really, really peed me off though, is the fact that nobody has said, I don’t have cancer”* (P34, no cancer found). This seemed to be influenced by a number of factors, including how satisfied they were with the further testing they had, communication from the consultant, plans for surveillance and safety netting advice (i.e. knowing what to look out for). Some participants in their 70 s interpreted the experience in relation to their age, describing acceptance of uncertainty about their future health: *“And when you’re in the 70 s, you start to think, well, anything can happen really, it’s only time, can’t it? So I’m not going to be surprised by anything”* (P29, no cancer found).

##### Meaning of the result

For those with no cancer found, questions about the meaning of the result revolved around why a signal was detected and their future risk of cancer. These questions were described as ‘in the back of my mind’ and the answers would ‘make me feel settled’ but most participants did not describe feeling worried: *“I suppose there is a question in the back of my mind. But it is right in the back of my mind as to, Okay, am I going to be developing a detectable [cancer] that they can’t detect yet. But I can’t say I’m worrying about that” (P28, no cancer found)*. Most acknowledged that they knew clarity about the meaning of results was not available (given this was a trial) e.g. “*I don’t think anybody would know that answer and presumably that may be part of your research” (P33, no cancer found)*.

## Discussion

Our study is the first to explore how people drawn from the general population interpret a cancer signal result in the context of an MCED screening test. For most participants the result was unexpected, and cancer was not something they had considered. This is consistent with previous research suggesting cancer screening participation is driven by a desire to look after health more generally [9] or because this fits with normative expectations [21], rather than thinking cancer is a possibility. This may have been exacerbated by the trial context. Many of the participants described the ‘bigger picture’ as their motivation for participation. They reflected on the opportunity to help ‘the next generation’, considering trials to be important rather than thinking about the possibility they might receive a diagnosis themselves. Our findings reflected conceptualisations of ‘lay epidemiology’ with participants constructing their perceived cancer risk based on family history, environmental factors and the symptomatic experience [22, 23], using these to make sense of the result.

Some participants were “matter of fact” about the experience and did not think ahead to a potential cancer diagnosis. Others were incredibly concerned that they may have cancer and visualised what this would mean for them. For some, the cancer signal result was interpreted as a cancer diagnosis. Previous work in cervical screening suggests the period between referral and follow-up appointments can bring confusion and concern, as well as the potential for considering “worst-case scenarios” [24, 25]. Our findings suggest that more intensive personalised support could be needed for some patients who receive a cancer signal result and require further testing. This support might include discussing worries about the potential for cancer, as well as decisions about sharing details of their result with others. Work in lung screening suggests that the opportunity for discussion of abnormal results can reduce psychological burden [26]. In the NHS-Galleri trial, participants were able to access support from the research nurses. This was described positively, as a “reassuring voice” by those who used it and having “someone you could talk to if you wanted” was valued even by those who did not.

For those who had been diagnosed with cancer by the time of interview, there seemed to be two types of response based on expectations. The first was characterised by surprise, following continued expectation that this would not be the case. This is consistent with work that shows the experience of a cancer diagnosis following screening can be jarring, perceived as a ‘whirlwind’ experience and acceptance can come later in the process than would be the case for symptomatic patients [12]. A second group of participants described being unsurprised when told they had cancer because the experience of having a cancer signal detected and going for further tests meant they had gradually come to terms with cancer as a possibility, often following ‘clues’ in the process. Narratives from these participants reflect what Locock et al. (2016) described as ‘the diagnostic assemblage’, highlighting how breaches, cues and clues throughout the patient journey can contribute to diagnostic experience and expectations [27].

Among those who had not been diagnosed with cancer at the time of interview, it was clear that some did not feel reassured and continued to worry that they did have cancer or that it may be found in the coming years. This echoes research in cervical screening that suggests for some a normal colposcopy does not provide reassurance following abnormal cytology [28] and with people who experience a false-positive FIT where some described not being able to ‘let go of the thought that something might still be wrong’ [29]. A recent review of women experiencing a false-positive breast screening, suggested that residual worry about cancer remains when inadequate explanations are given for the result [10]. Since our study was embedded in a trial, it was not possible to provide explanations for the scenario where a cancer signal is detected but no cancer subsequently found. If MCED screening is implemented in the future, providing answers to questions about the meaning of the result will be vital.

Participants’ expectations of cancer played an important role all the way through the diagnostic process and influenced thoughts about the future, emotional reactions and social experiences. This is consistent with the cognitive principle, i.e. a person’s emotional and behavioural responses follow their thoughts about an event [30]. If thoughts in the face of a cancer signal result follow the thread of expectations, we can design support around this. At its most basic level, this might mean acknowledging expectations about the result and its meaning in result communications. More personalised support might be available to allow people to deconstruct and interpret the meaning of the cancer signal detected result, including the possible tissue type or organ associated with the signal, in light of their own personal history and beliefs. For some, who need more intense support, drawing on cognitive approaches (e.g. mindfulness, reframing thoughts, or Acceptance and Commitment Therapy) might help people to make sense of information and adapt to their result. This approach has recently been suggested for use in a genetic counselling context [31].

### Strengths and limitations

This is the first study to explore in-depth the experiences of people having a ‘positive’ result following an MCED test in a screening scenario. The size of the NHS-Galleri trial meant we were able to purposively sample and include a range of people based on sex, age and socio-economic background, as well as including those who went on to have a cancer diagnosis and those who did not. There were, however, some groups that were under-represented, particularly those from minoritised ethnic backgrounds. Given the novelty of MCEDs, a cancer signal detected screening result was completely new to people. Unlike existing screening programmes, no one had family or friends with similar experiences and there were no online support groups; something that is often used to find those with shared experience [32]. If MCED screening is offered routinely, people would be more able to share similar experiences and might make sense of their results in different ways. If MCED screening is implemented, additional research will therefore be needed to explore whether different themes arise. Participants were interviewed 6-months after their cancer signal was detected and at the point that they knew whether they had cancer or not. This time lapse and knowledge of the diagnostic outcome may have influenced the way participants retrospectively described their perceptions.

Psychological implications of having a signal detected and a cancer diagnosed will likely be influenced by the survival for that cancer and the treatment required. Participants’ clinical characteristics were diverse, with a broad range of CSOs communicated, follow-up tests required (including scans, biopsies and further blood tests), and cancers diagnosed (in those who received a diagnosis). While this is a strength in some respects because it ensures a wide range of perspectives, it means we cannot make inferences about the impact of experiencing particular outcomes or diagnostic tests/treatments. This is in some ways a challenge for research in the MCED space, where post-test trajectories have the potential to be very different. In addition, because of the inherent ‘survivor bias’ introduced by interviewing people 6 months after receiving their MCED results, we were not able to include the perspectives of anyone who died within 6 months of receiving their results.

## Conclusions

In some ways, having a cancer signal detected through an MCED test will be similar to having abnormal results in other screening programmes. However, there are novel aspects of MCED screening with which people experiencing a positive result may need support. This includes assimilating thoughts about the possibility of cancer, including where it could be in the body, in light of their personal health and family history. Helping people interpret a cancer signal detected result in light of other screening test results and make sense of the meaning if cancer is ultimately not found will also be important. There was clear variation in how people reacted to and made sense of their result, how worried they were about it, how they managed communicating with others, and how reassured they were if cancer was not found. While written information can be designed with these aspects in mind, it is likely that for some additional support will be needed and we recommend that this is seriously considered alongside decisions about a future implementation.

## Supplementary information

COREQ (Consolidated criteria for Reporting Qualitative research) Checklist

Reproducibility checklist

## Data Availability

The datasets generated during and/or analysed during the current study are not publicly available due to the need to protect individual privacy of participants.
